# *Kleinia* (Asteraceae): comprehensive review of ethnomedicinal uses, phytochemical profiles, ethnopharmacological applications, and toxicological insights

**DOI:** 10.3389/fphar.2024.1469887

**Published:** 2025-01-08

**Authors:** Bantayehu Addis Tegegne, Tesfa Begashaw, Wubetu Yihunie Belay, Mengistie Kassahun Tariku, Tirsit Ketsela Zeleke, Mohammed Jemal, Mamaru Getinet, Agumas Alemu Alehegn, Abebe Dagne

**Affiliations:** ^1^ Department of Pharmacy, College of Medicine and Health Sciences, Debre Markos University, Debre Markos, Ethiopia; ^2^ Department of Pharmacy, College of Medicine and Health Sciences, Debre Birhan University, Debre Birhan, Ethiopia; ^3^ Department of Public Health, College of Medicine and Health Sciences, Debre Markos University, Debre Markose, Ethiopia; ^4^ Department of Biomedical Sciences, School of Medicine, Debre Markos University, Debre Markose, Ethiopia; ^5^ Department of Pharmacy, Amhara Regional State Public Health Institute, Bahir Dar, Ethiopia

**Keywords:** *Kleinia*, ethnomedicinal uses, phytochemicals, metabolites, ethnopharmacology, toxicology

## Abstract

*Kleinia* is a genus of over 50 species that are commonly used in primary care in several countries. This study seeks to inspire researchers to quickly discover and isolate the key active metabolites found in *Kleinia* taxa, thereby promoting the development of novel, safe, and effective therapies for a variety of illnesses. To this end, we performed a thorough search of English-language publications from PubMed, Scopus, ScienceDirect, Web of Science, Google Scholar, and ResearchGate. Our search utilized keywords such as “ethnobotany,” “geographic distribution,” “ethnomedicinal use,” “phytochemistry,” “pharmacological or bioactivities,” and “toxicological activities” related to the genus *Kleinia*. Chemical structures were depicted using Chemdraw^®^ software. Literature highlights numerous *Kleinia* taxa used in traditional medicine for conditions like intestinal parasites, measles, smallpox, diabetes, edema, nerve disorders, sexual dysfunction, gastrointestinal issues, cancer and more. Phytochemical analysis identifies 77 secondary metabolites, mainly alkaloids, flavonoids, saponins, terpenes, and terpenoids and other miscellaneous metabolites. Among the *Kleinia* taxa, *K. anteuphorbium*, *K. longiflora*, *K. grandiflora*, *K. odora*, *K. squarrosa*, *K. abyssinica*, *K. pendula*, and *K. azoides* have been scientifically validated to exhibit various pharmacological activities. However, the existence of potentially harmful metabolites in *Kleinia* taxa, particularly pyrrolizidine alkaloids, emphasizes the significance of cautious application in traditional medicine and the need for rigorous toxicological assessments. In conclusion, this review highlights the promise of *Kleinia* taxa as significant medicinal resources and advocates for extensive bioprospecting. It encourages global pharmaceutical companies and academic institutions to conduct thorough investigations of the genus *Kleinia* to uncover new therapeutic possibilities.

## 1 Introduction

At least 80% of people worldwide now rely on herbal supplements and pharmaceutical goods for some aspect of their basic healthcare, a huge growth in use over the previous three decades. Even though treatments incorporating these substances have demonstrated encouraging promise and the effectiveness of many herbal products has been established, many of them are still untested, and their usage is either not tracked at all or is only partially monitored (Ekor, 2014).

Since ancient times, medicinal plants have played a crucial role in treating ailments and have been integral to human society. Evidence from various sources, such as written records, monuments, and preserved plant medicines, attests to humanity’s long history of seeking remedies in nature. The utilization of plants for therapeutic purposes is a practice that has a history spanning 3,000 years, with noteworthy figures such as Abu Rayhan, Biruni, Ibn Baytar and Ibn Sina making significant contributions to the field of herbal medicine. Humanity’s knowledge of medicinal plants developed through centuries of battling illnesses, learning to harness the healing properties of leaves, bark, seeds, and other plant parts. Global research continues to validate their effectiveness, leading to the creation of plant-based medicines (Schaal, 2019; Sofowora et al., 2013; Petrovska, 2012; Akbulut et al., 2019).

The World Health Organization (WHO) estimates that 4 billion people, or 80% of the world’s population, currently obtain basic healthcare through herbal medicine (Bodeker and Ong, 2005). The effectiveness, cost, cultural relevance, and lack of modern medical facilities make it believed that 80% of people in developing countries rely on traditional herbal medications for their primary healthcare. Likewise, in affluent nations, 80% of people are using traditional medicines, which are quickly increasing in use due to their cultural acceptance, affordability, and restricted access to contemporary medications. Plants have long played a significant role in human culture, beliefs, and traditional medicine. Plant-based medicine has been a crucial part of treatment since ancient times. With between 50,000 and 75,000 plant species utilized in both traditional and modern medicine, the majority of people on the planet, particularly in underdeveloped countries, rely on plants for both sustenance and medical care. In addition to producing a vast range of organic substances, plants are used for a wide range of purposes, such as food, medicine, shelter, dyes, healing, religious ceremonies, clothing, and decoration (Aziz et al., 2018a; Şen et al., 2022; Aziz et al., 2018b).

Medicinal plants provide bioactive metabolites that are valuable for human pharmacology. Therefore, to acknowledge the significance of traditional medicine in national healthcare, governments should gather data on medicinal plant species and their traditional uses. They should also integrate traditional medicine into the healthcare system by establishing supportive administrative structures for its practice (Smith-Hall et al., 2012; Usure et al., 2024; Asong et al., 2019).

According to the International Union for Conservation of Nature (IUCN) and the World Wildlife Fund (WWF), there are between 50,000 and 80,000 flowering plant taxa used for medicinal purposes worldwide. Among these, about 15,000 plant taxa are threatened with extinction from overharvesting and habitat destruction, and nearly exhausted with the increasing human population and plant consumption. Although this threat has been known for decades, the accelerated loss of species and habitat destruction worldwide has increased the risk of extinction of medicinal plants, especially in China, India, Nepal, Kenya, Tanzania Uganda, and Ethiopia (Chen et al., 2016).

Among plant taxa worldwide, 50 belong to the genus *Kleinia*, which are used in traditional medicine. In Ethiopia, four species from this genus have specific uses: *Kleinia odora* DC. (so called Luko in Afaan Oromo) is traditionally used for nerve-related issues, *Kleinia abyssinica* (A.Rich.) A. Berger (also known as Abrasha in Afaan Oromo) for sexual dysfunction, *Kleinia pendula* DC. (Afrasha in Afaan Oromo) for treating swollen body parts, and *Kleinia squarrosa* Cufod. (also called Luko, sharing the vernacular name with *Kleinia odora* DC.) for intestinal parasites (Bussa and Belayneh, 2014; Ouhaddou et al., 2014).

Few *Kleinia* taxa have been the subject of numerous *in vitro* and/or *in vivo* scientific papers, but there isn't a thorough analysis that combines the various therapeutic uses of this genus worldwide. In order to achieve this, this review will examine the phytochemical metabolites, pharmacological characteristics, toxicological safety, and traditional medicinal uses of *Kleinia* (Asteraceae). It will also draw attention to the need for additional research on species that have not yet been studied and for expanding studies on species that have been studied in clinical settings.

## 2 Methodology

### 2.1 Literature searching strategy

An extensive literature search was conducted to gather relevant information on ethnomedicinal uses, geographic distribution, phytochemistry, pharmacology, and toxicology. This process involved reviewing a wide range of scientific sources, including articles, book chapters, books, and encyclopedias written in English and supported by scientific research. The key search terms used included: “ethnomedicinal uses”,“geographic distribution”, “phytochemistry”, “pharmacological activities”,” toxicological safety”.

### 2.2 Databases searched

The literature search spanned several major scientific electronic databases to ensure comprehensive coverage of the subject. Relevant articles, book chapters, books, and encyclopedias have been extensively searched from PubMed, Scopus, Springer Link, Sci-Finder, Science Direct, Google Scholar, and Research Gate. Each database was searched using a combination of the identified keywords, and results were reviewed to identify studies and materials that aligned with the research objectives.

### 2.3 Inclusion criteria

The following criteria were used to select literature: Publications written in english; research backed by scientific evidence; works that aligned with the study’s focus on ethnomedicine, phytochemistry, pharmacology, and toxicology; and preference was given to recent studies, but seminal works were also included when relevant.

### 2.4 Data extraction

From the selected literature, key data was extracted, focusing on the following: Ethnomedicinal uses (traditional medicinal applications of the plant taxa); geographic distribution (regions where the plants are native or commonly found); phytochemistry (identification and analysis of chemical metabolites found in the plant species); pharmacological effects (documented therapeutic benefits and biological activities); toxicological properties (information on the safety profile, side effects, and toxicological studies).

### 2.5 Structural identification of phytochemicals

The chemical structures of metabolites identified in the phytochemical analysis were displayed using Chemdraw^®^ software. This allowed for clear visualization of the molecular structures and ensured accurate representation of the phytochemical data.

### 2.6 Data analysis and synthesis

After the extraction, the data was organized and synthesized to present a clear understanding of the ethnomedicinal relevance, and chemical composition of the plants. Comparative analysis was also conducted to correlate phytochemistry with pharmacological and toxicological findings, ensuring an integrated approach to understanding the medicinal value of the *Kleinia*.

## 3 The genus *Kleinia (*Asteraceae): geographical distribution and morphology

*Kleinia* belongs to the Asteraceae family, a group of flowering plants (angiosperms). The genus is named after 18th-century German botanist Dr. Jacob Theodor Klein (Halliday, 1984a). It is an annual succulent shrub, with striking, thin, pencil-shaped, segmented, and upright or sprawling stems, that is drought resistant (Smith, 2011). The genus *Kleinia* is distributed through the Atlantic Ocean, Tropical Africa, Subtropical Africa, India, and Arab countries. It is found in cold to temperate climates at altitudes of 300–1,600 m with different habitats of rocks, grassland, and woodland (Mies, 1995; Halliday, 1984b). Table 1 summarizes the geographic distribution of a few common taxa under the *Kleinia*.

**TABLE 1:** Distribution patterns of thirteen documented *Kleinia* taxa.

Species name	Geographical distribution	Reference
*Kleinia odora* (Forssk.) DC.	Somalia, Ethiopia, Djibouti, Saudi Arabia, Sudan, (North and South), Uganda, Kenya, Tanzania	Al-Namazi et al. (2021), Al Musayeib et al. (2013)
*Kleinia deflersii (O.Schwartz)* P.Halliday	Saudi Arabia	Al Musayeib et al. (2013)
*Kleinia grandiflora* DC.	Thailand	Vanijajiva et al. (2014)
*Kleinia anteuphorbium* (L.) DC.)	Morocco	Elhidar et al. (2019)
*Kleinia longiflora* DC.	South Africa, Namibia, Botswana, Swaziland	Semenya and Maroyi (2018)
*Kleinia breviflora* C.Jeffrey	Kenya, Tanzania, Thailand	Vanijajiva et al. (2014), Clayton et al. (1982), Rowley and Eggli (2002)
*Kleinia neriifolia* Haw	Spain, North Africa, Southern Arabian Peninsula, South Africa, Madagascar	Sun and Vargas-Mendoza (2017), Henderson (2006)
*Kleinia venteri* Van Jaarsv	South Africa, South America (Uruguay, Argentina, Chile, South Brazil), New Zealand, Australia	Henderson (2006), Serumula (2021)
*Kleinia petraea* (R.E.Fr.) C.Jeffrey	South Africa, Brazil (naturally). Moreover, Paris, France (herbarium specimens)	Melo-de-Pinna et al. (2016)
*Kleinia saginata* P.Halliday	South Africa, Brazil (naturally). Moreover, Paris, France (herbarium specimens)	Melo-de-Pinna et al. (2016)
*Kleinia squarrosa* Cufod	Ethiopia, Djibouti, Uganda, Kenya, Tanzania, and South Arabia	Jeffrey (1986), Guta et al. (2018)
*Kleinia pendula (Forssk.)* DC	Ethiopia, Eretria, Kenya, Somalia, Saudi Arabia, Yemen, Philips	Al Musayeib et al. (2013), Ozerova et al. (2017), Sewawa (2021)
*Kleinia abyssinica* (A.Rich.) A.Berger	Ethiopia, Burundi, Kenya, Ruanda, Tanzania, Uganda, Netherlands, South Sudan, Malawi, Zambia, Democratic-Republican Congo, Cameroon	Lulekal et al. (2008), Chekole (2017), Mokua et al. (2021), Ouhaddou et al. (2014)

**FIGURE 1:**
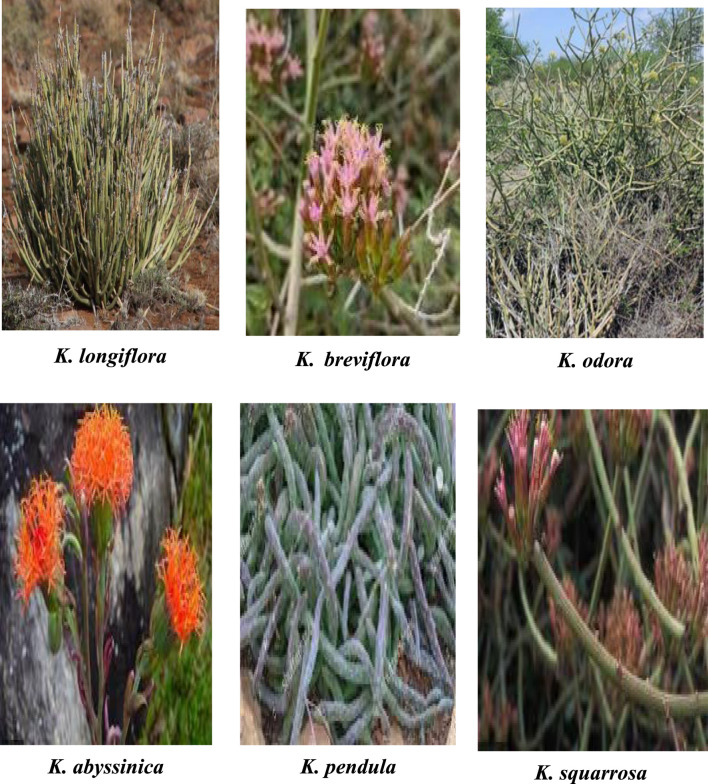
Some picture of a few *Kleinia* taxa.

The *Kleinia* genus consists of perennial flowering plants characterized by succulent leaves, which can be either flat or cylindrical. These plants typically have angled, fleshy stems and may possess tuberous roots. Their leaves and flowers are usually smooth, with flower heads that vary in size, containing multiple disk-shaped, often uniform flowers, though some species have mixed types. The flowers come in white, yellow, or red, with a flat receptacle. The genus features conical, papillose appendages, and the fruit is non-beaked, cylindrical, ribbed, and can be either smooth or hairy. The genus *Kleinia* consists of upright, perennial subshrubs that grow between 1–2.5 m tall, with stems measuring 3–6 cm in diameter. The plants have petioles ranging from 2–4 cm in length and flower heads that are 1.9–2 cm long and 2–2.5 cm wide. The floral lobes are at least 3 mm long, while the anthers measure 4 mm in length (Halliday, 1984b; Vanijajiva et al., 2014; Melo-de-Pinna et al., 2016; Lulekal et al., 2008; Timonin et al., 2014; Cicuzza et al., 2018). Figure 1 displays a few *Kleinia* taxa.

## 4 Ethnomedicinal use of the genus *Kleinia*

As displayed Table 2, traditional medicine in Ethiopia, Morocco, South Africa, South India, China, and North America has utilized different parts of numerous *Kleinia* taxa to treat a range of human diseases and conditions.

**TABLE 2:** Ethnobotanical medicinal use of *Kleinia* taxa.

S/N	Species	Part(s) utilized	Mode of preparation	Route	Disease Treated	Countries report	Reference
1	*K. odora* *K. abyssinica* *K. pendula* *K. squarrosa*	Leaves Rhizome Stem Stem	By boiling and mixing with other plant Fresh rhizome few h before Sexual Performance Via bandaging the body with a fresh decoction of succulent stem By taking the crushed stem	Oral Oral Topical Oral	Nerve cases Sexual dysfunction Swollen body Intestinal parasite	Ethiopia	Belayneh and Bussa (2014)
2	*Kleinia anteuphorbium* (L.) DC *Kleinia anteuphorbium* (L.) DC	Stem Leaves	Via the external application of the juice, powder, and infusion preparation The essential oil extracted from the leaves	Topical	respiratory, skeleton, and circulatory complication as a sedative for abdominal/back pain, anti-inflammatory, and rheumatism	Morroco	Ouhaddou et al. (2014)
3	*K. grandiflora*	Leaves With roots Leaves	Extracts of leaves with roots 2 or 3 drops of fresh leaf juices	Orally Via ear	gastric complaints, inflammatory disease, and analgesic activity earache	South India	Ju et al. (2019)
4	*K. longiflora*	Root	Decoction product obtained from the root Macerated product of the root of *K. longiflora* and Decoction of the root with warm water for 24 h is administered	Orally Rectally	Sexual transmitted infection Menstrual disorders	South Africa	Maroyi (2014)
5	*Kleinia aizoides* DC.	Leaves	The extracts of the fresh leaf, as well as the dried leaves The extract of the dried powdered leaves is dropped three times per day	Orally Ocular	Internal bleeding and reduction of blood pressure eye infection	North America	Maroyi (2014)

## 5 Phytochemical composition of the genus *Kleinia*

An overview of the works of literature search indicated that some *Kleinia* taxa have been investigated phytochemically from 50 plant species known from this genus namely, *K. odora* (Shehata et al., 2018), *K. squarrosa* (Juma et al., 2015)*, K. pendula* (Alfaifi et al., 2020)*, K. longiflora* (Sewawa, 2021)*, K. grandiflora* (Pitchaipillai et al., 2014; Selvakumar, 2018)*, K. anteuphorbium* (Aati et al., 2024a)*,* and *K. aizoide* (Nunez‐Melendez and Johnson, 1955). This genus has a phytochemical of flavonoids (19), saponins (6), cardiac glycosides (8), alkaloids (20), terpenes (8), terpenoids (5), and other types of metabolites (11) have been isolated and identified from the above-listed species.

### 5.1 Flavonoids

Flavonoids, a class of natural metabolitess with varying phenolic structures, are found in fruits, vegetables, cereals, bark, roots, stems, flowers, tea, and wine. These natural products are well-known for their health benefits, and efforts are underway to isolate the metabolites known as flavonoids. Flavonoids have a variety of medicinal properties, including anticancer, antioxidant, anti-inflammatory, and antiviral activity. They also have neuro-protective and cardio-protective properties. These biological activities depend upon the type of flavonoid, its mode of action, and its bioavailability (Panche et al., 2016; Ullah et al., 2020).

As shown in Table 3, a total of 19 flavonoids are isolated from *Kleinia* taxa like *K. grandiflora, K. longiflora K. anteuphorbium, K. grandiflora, K. odora, K. aizoides.* These are flavanone (1), flavone (8), flavonol (3), dihydroflavonol (3), and anthocyanidin (4).

**TABLE 3:** Flavonoids isolated from *Kleinia* taxa aerial parts.

No.	Name of the metabolites	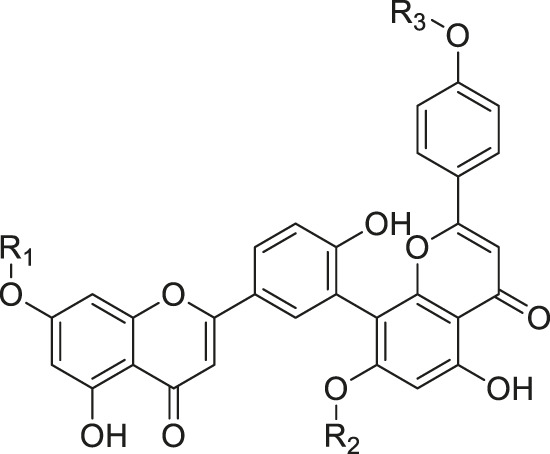				Species	References
		R1	R2	R3	Class of metabolites		
1	Heveaflavone	CH3	CH3	CH3	Biflavonoid	*K. odora* *K. squarrosa*	Shehata et al. (2018), Juma et al. (2015)
2	Amentoflavone −7,4-dimethyl Ether	H	CH3	CH3	Biflavonoid	*K. anteuphorbium* *K. longiflora* *K. grandiflora* *K. odora*	Aati et al. (2024a), Sewawa (2021), Pitchaipillai et al. (2014), Selvakumar (2018), Shehata et al. (2018)
3	Podocarpus flavone	H	H	CH3	Biflavonoid	*K. pendula*	Alfaifi et al. (2020)
		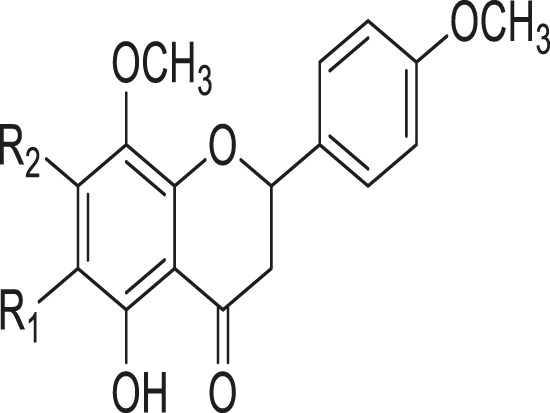					
		R1	R2		Class of Metabolites		
4	5,7-dihydroxy-4,6,8-trimetoxyflavone	OMe	OH		Flavanone	*K. grandiflora* *K. odora* *K. squarrosa* *K. aizoides*	Pitchaipillai et al. (2014), Selvakumar (2018), Shehata et al. (2018), Juma et al. (2015), Nunez‐Melendez and Johnson (1955)
5	5,6-dimethoxy 4,7,8-trimetoxyflavone	OH	OMe		Flavanone	*K. squarrosa* *K. pendula*	Juma et al. (2015), Alfaifi et al. (2020)
		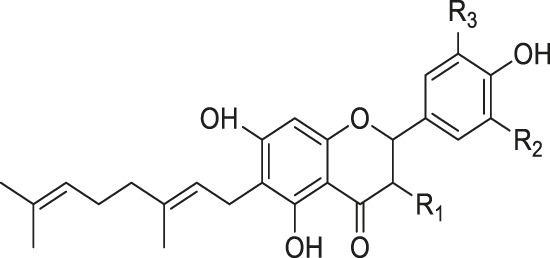					
		R1	R2	R3	Class of Metabolites		
6	3-O- methyl-5-hydroxy diplacone	H	OMe	OH	Flavanone	*K. grandiflora* *K. odora*	Pitchaipillai et al. (2014), Selvakumar (2018), Shehata et al. (2018)
7	3-O-methyl-5-O-methyl diplacone	H	OMe	OMe	Flavanone	*K. anteuphorbium* *K. longiflora*	Pitchaipillai et al. (2014), Selvakumar (2018), Sewawa (2021)
8	Mimulone	H	H	H	Flavone	*K. pendula*	Alfaifi et al. (2020)
9	Diplacone	H	OH	H	Flavone	*K. squarrosa*	Juma et al. (2015)
		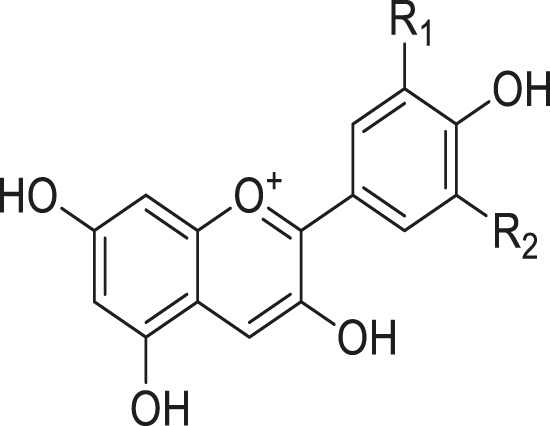					
		R1	R2		Class of Metabolites		
10	Delphinidin	OH	OH		Anthocyanidin	*K. anteuphorbium* *K. longiflora* *K. odora*	Aati et al. (2024a), Sewawa (2021), Shehata et al. (2018)
11	Pelargonidin	H	H		Anthocyanidin	*K. anteuphorbium*	Aati et al. (2024a)
12	Peonidin	OCH3	H		Anthocyanidin	*K. longiflora* *K. grandiflora* *K. odora*	Sewawa (2021), Pitchaipillai et al. (2014), Selvakumar (2018), Shehata et al. (2018)
13	Malvidin	OCH3	OCH3		Anthocyanidin	*K. squarrosa*	Juma et al. (2015)
		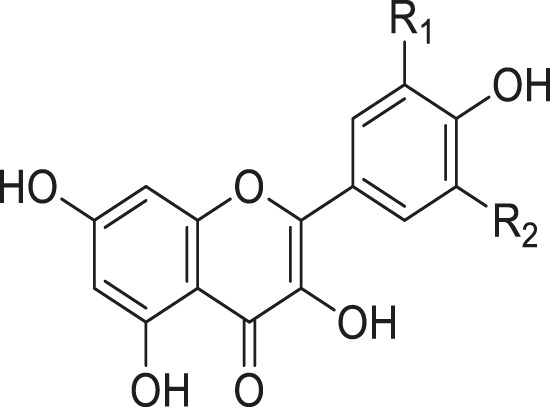					
		R1	R2		Class of Metabolites		
14	Kaempferol	H	H		Flavonol	*K. odora* *K. squarrosa*	Shehata et al. (2018), Juma et al. (2015)
15	Quercetin	OH	H		Flavonol	*K. anteuphorbium* *K. longiflora* *K. grandiflora* *K. odora*	Aati et al. (2024a), Sewawa (2021), (Pitchaipillai et al. (2014), Selvakumar (2018), Shehata et al. (2018)
16	Myricetin	OH	OH		Flavonol	*K. anteuphorbium* *K. longiflora* *K. grandiflora* *K. odora*	Aati et al. (2024a), Sewawa (2021), Pitchaipillai et al. (2014), Selvakumar (2018), Shehata et al. (2018)
		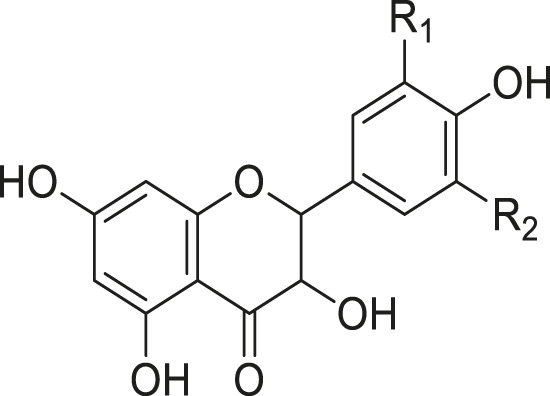					
		R1	R2		Class of Metabolites		
17	Dihydrokaempeferol	H	H		dihydroflavonol	*K. longiflora* *K. grandiflora* *K. odora*	Sewawa (2021), Pitchaipillai et al. (2014), Selvakumar (2018), Shehata et al. (2018)
18	Diyhydroquercetin	OH	H		dihydroflavonol	*K. longiflora* *K. grandiflora* *K. odora*	Sewawa (2021), Pitchaipillai et al. (2014), Selvakumar (2018), Shehata et al. (2018)
19	Dihydromyricetin	OH	OH		dihydroflavonol	*K. longiflora* *K. grandiflora* *K. odora*	Sewawa (2021), Pitchaipillai et al. (2014), Selvakumar (2018), Shehata et al. (2018)

### 5.2 Saponins

Since their biodiversity is still mostly unknown, saponins are one of the most prevalent and varied families of plant natural products. They also hold considerable promise for future medication discovery. Their hydrophilic carbohydrate chain and hydrophobic triterpene or sterol backbone are what give them their amphipathic character and biological activity. But other structural metabolites also play a part in their powerful impacts. Saponins are naturally occurring secondary metabolites that are amphiphilic, glycosidic, and heat-stable. Because of their triterpenoid or steroidal aglycones connected to oligosaccharides, saponins are used extensively in the pharmaceutical sector (Mugford et al., 2013; Yang et al., 2021).

Saponins isolated from the species of genus *Kleinia* are summarized in Table 4. There are two major classes of saponins isolated from the genus *Kleinia* namely; steroidal saponin and triterpenoid saponin. Protopanaxadio (20), protopanaxatriol (21), gypsogenin (22), gypsogenic acid (23), quillaic acid (24), and hederagenin (25) are some examples of metabolites isolated from those major classes of saponins (Jesus et al., 2015).

**TABLE 4:** Saponins isolated from *Kleinia* taxa aerial part.

No.	Name of the metabolites	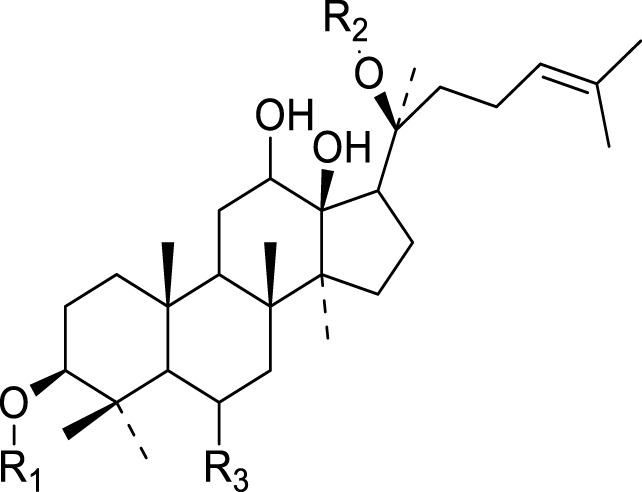				Species	Reference
		R1	R2	R3	Class of metabolites		
20	Protopanaxadiol	Sugar (Glucose)	Sugar (Glucose)	H	Steroidal saponin	*K. anteuphorbium* *K. odora* *K. squarrosa*	Aati et al. (2024a), Shehata et al. (2018), Juma et al. (2015)
21	Protopanaxatriol	H	Sugar (Glucose)	O-sugar	Steroidal saponin	*K. odora* *K. squarrosa* *K. pendula* *K. aizoides*	Shehata et al. (2018), Juma et al. (2015), Alfaifi et al. (2020), Nunez‐Melendez and Johnson (1955)
		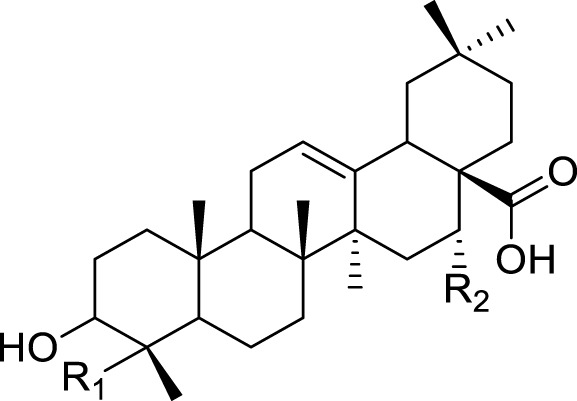					
		R1	R2	Class of Metabolites			
22	Gypsogenin	CHO	H	Triterpenoid saponin		*K. longiflora* *K. grandiflora*	Sewawa (2021), Pitchaipillai et al. (2014), Selvakumar (2018)
23	Gypsogenic acid	COOH	H	Triterpenoid saponin		*K. odora* *K. squarrosa*	Sewawa (2021), Juma et al. (2015)
24	Quillaic acid	CHO	OH	Triterpenoid saponin		*K. longiflora* *K. pendula*	Sewawa (2021), Alfaifi et al. (2020)
25	Hederagenin	CH2OH	H	Triterpenoid saponin		*K. anteuphorbium* *K. odora* *K. squarrosa*	Aati et al. (2024a), Sewawa (2021), Juma et al. (2015)

### 5.3 Alkaloids

Alkaloids are nitrogen-containing metabolites characterized by a heterocyclic ring structure composed of hydrogen and carbon, sometimes including sulfur and oxygen. According to studies in natural products chemistry, alkaloids are naturally occurring substances that can be extracted from the crude extracts of bacteria, fungi, plants, and animals. In plants, alkaloids exhibit diverse structural forms, which are classified into three categories: protoalkaloids, pseudoalkaloids, and true alkaloids. The bioactivity of each group is influenced by the complexity of its structural backbone, with amino acids, which make up 80% of these backbones, playing a crucial role in the biosynthesis of alkaloids. Alkaloids have several uses in medicine and other facets of human life, such as medications, dietary additives, and supplements (Bhambhani et al., 2021; Joanna and Joanna, 2019).

**FIGURE 2:**
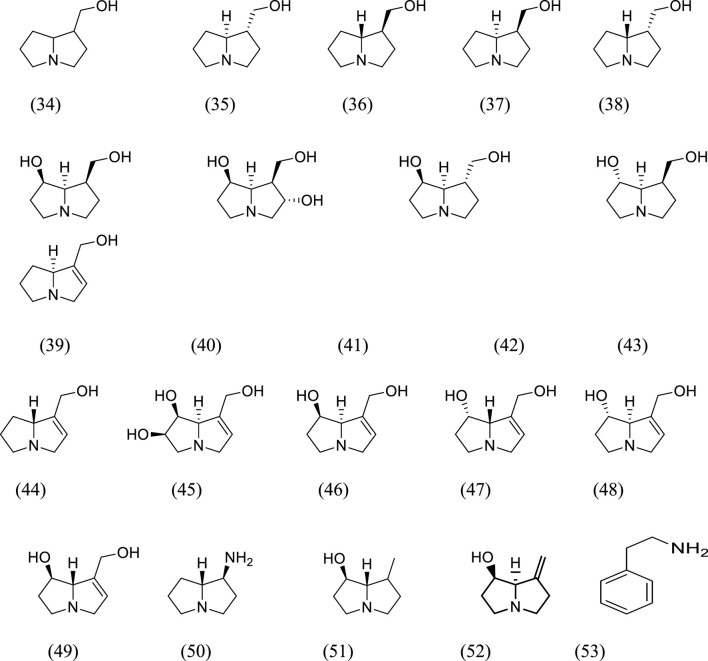
The structural formula of the alkaloids that were separated from *Kleinia* taxa.

Pyrrolizidine and cyclic amine alkaloids isolated from the species of genus Kleinia (Figure 2) are 1-hydroxymethylpyrrolizidine (34), (−)trachelanthamidine (35), (+) trachelanthamidine (36), (−) isoretronecanole (37), (+)-isoretronecanole (38), (−)-platynecine (39), (−)-rosmarinecine (40), (−)-tumeforcidine (41), (−)-dihydroheliotridane (42), (−)-supinidine (43), (+)-supinidine (44), (−)-crotanecine (45), (+)-retronecine (46), (−)-retronecine (47), (+)-heliotridine (48), (1S,8R)-1-aminopyrrolizidine (49), (1R,7S)-7-methylhexahydro-1H-pyrrolizine-1-ol (50), (1R,7R)-7-methylidenehexahydro-1H-pyrrolizin-1-ol (51), (7S,8R)-petranecine (52), and phenyl ethyl amine (Romo de Vivar et al., 2007).

### 5.4 Terpenes and terpenoids

The primary bioactive metabolites, of essential oils are terpenes and terpenoids. Terpenes, such as pinene, myrcene, limonene, terpinene, and p-cymene, are metabolites composed of simple hydrocarbon structures, while terpenoids are a modified class of terpenes that contain oxygen and have altered functional groups, including oxidized or repositioned methyl groups. Terpenes are known to exhibit antimicrobial activity against both antibiotic-susceptible and antibiotic-resistant bacteria, primarily by promoting cell membrane disruption and inhibiting protein and DNA synthesis. Terpenoids are divided into monoterpenes, sesquiterpenes, diterpenes, sesterpenes, and triterpenes based on the number of carbon units contained in them (Masyita et al., 2022; Shagufta et al., 2018; Efe et al., 2021).

**FIGURE 3:**
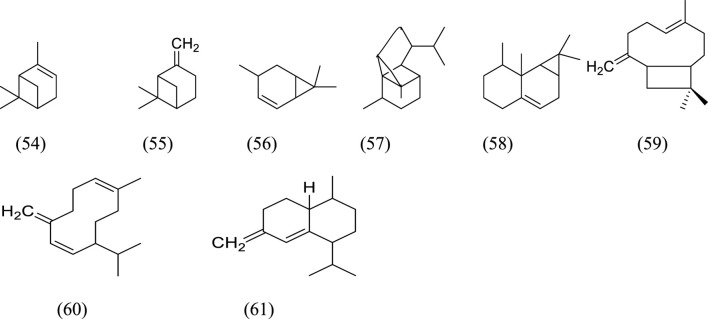
Structural formulas of terpenes isolated from *Kleinia* taxa.

Monoterpenes and sesquiterpenes isolated from genus *Kleinia* (Figure 3) are-α-pinene (54), β-pinene (55), (+)-4-carene (56), copaene (57), aristolene (58), α-caryophyllene (59), germacrene-D (60), and (+)-Epi-bicyclosesquiphellandrene (61) are isolated from the species *K. anteuphorbium, K. longiflora, K. grandiflora, K. odora, K. squarrosa, K. abyssinica, K. tomentosa, K. aizoides,* and *K. pendula*. Triterpenoids like ursolic acid (55), uvaol (56), hydroxyursolic acid (57), trans-cinnamoyloxy-hydroxyurs-12-en-28-oic acid (58), and trans-p-coumaroyloxy-hydroxyurs-12-en-28-oic acid (59) are isolated mainly from the species *K. anteuphorbium, K. longiflora, K. grandiflora, K. odora, K. squarrosa, K. aizoides,* and *K. pendula* (Al Musayeib et al., 2013; Efe et al., 2021; Migahid, 1988; Bohlmann and Knoll, 1978; Bohlmann and Suding, 1980; Bohlmann et al., 1981; Al-Taweel et al., 2004; Al-Taweel et al., 2001; Mohanraj et al., 2018). Table 5 below summarizes the main triterpenoids isolated.

**TABLE 5:** Triterpenoids isolated from *Kleinia* taxa aerial parts.

No.	Name of the metabolites	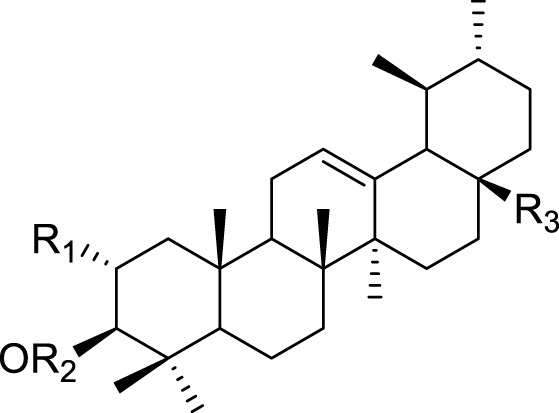				Species	Reference
		R1	R2	R3	Class of metabolites		
62	Ursolic acid	H	H	COOH	Triterpenoid	*K. pendula* *K. aizoides*	Alfaifi et al. (2020), Nunez‐Melendez and Johnson (1955)
63	Uvaol	H	H	CH2H	Triterpenoid	*K. anteuphorbium* *K. longiflora* *K. grandiflora* *K. odora* *K. squarrosa*	Aati et al. (2024a), Sewawa (2021), Pitchaipillai et al. (2014), Selvakumar, 2018, Shehata et al. (2018), Juma et al. (2015)
64	hydroxyursolic acid	OH	H	COOH	Triterpenoid	*K. grandiflora* *K. odora* *K. squarrosa*	Pitchaipillai et al. (2014), Selvakumar (2018), Shehata et al. (2018), Juma et al. (2015)
65	trans-cinnamoyloxy-hydroxyurs-12-en-28-oic acid	OH	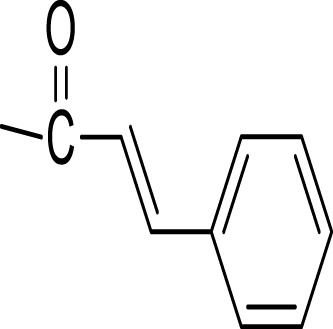	COOH	Triterpenoid	*K. anteuphorbium* *K. longiflora* *K. pendula*	Aati et al. (2024a), Sewawa (2021), Alfaifi et al. (2020)
66	Trans-p-coumaroyloxy-hydroxyurs-12-en-28-oic acid	OH	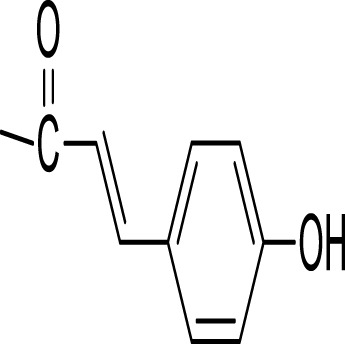	COOH	Triterpenoid	*K. anteuphorbium* *K. longiflora* *K.grandiflora* *K. odora* *K. pendula*	Aati et al. (2024a), Sewawa (2021), Pitchaipillai et al. (2014), Selvakumar (2018), Shehata et al. (2018), Alfaifi et al. (2020)

### 5.5 Miscellaneous metabolites isolated from the genus *Kleinia*

**FIGURE 4:**
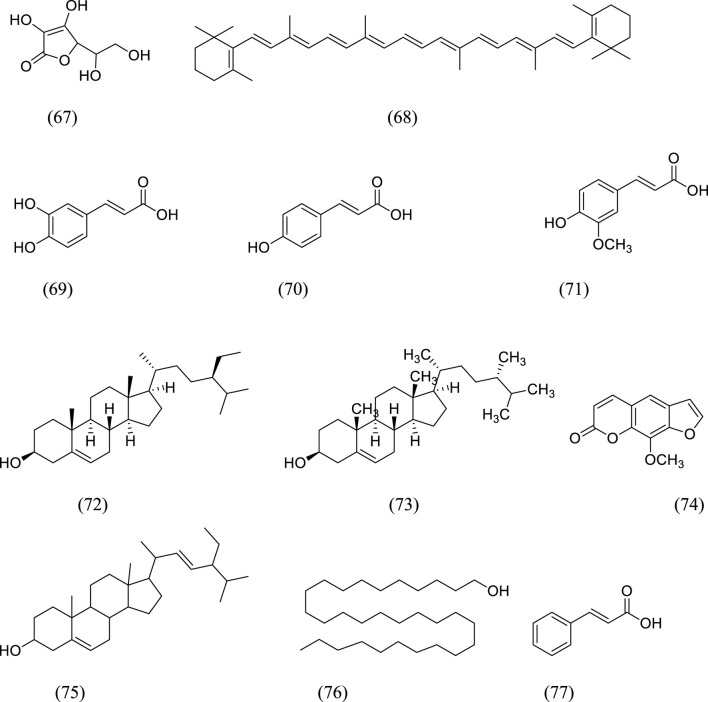
The structural formula of miscellaneous metabolites isolated from *Kleinia* taxa.

Figure 4 shows various chemicals that have been isolated from the genus *Kleinia*. Ascorbic acid (67), β-carotene (68), caffeic acid (69), P-coumaric acid (70), ferulic acid (71), β-sitosterol (72), campesterol (73), xanthotoxin (74), stigmasterol (75), n-triacontanol (76), and cinnamic acid (77) are isolated from the species *K. anteuphorbium, K. longiflora, K. grandiflora, K. odora, K. squarrosa, K. abyssinica, K. azoides, and K. pendula* (Jeffrey, 1986).

## 6 Pharmacological and toxicological studies from the genus *Kleinia* taxa

### 6.1 Pharmacological studies from the genus *Kleinia* taxa

Pharmacological studies on various *Kleinia* species underscore their importance as medicinal plants. A detailed review of the literature reveals specific pharmacological reports for *K. anteuphorbium, K. longiflora, K. grandiflora, K. odora, K. squarrosa, K. abyssinica, K. pendula,* and *K. azoides*. These studies highlight a wide range of therapeutic properties, including analgesic, anti-inflammatory, antipyretic, anticancer, antiprotozoal, antidiabetic, antimicrobial, and antifungal effects, supported by both *in vitro* and *in vivo* experimental evidence.

However, the mechanisms of action underlying these effects remain largely unexplored, and there is a notable absence of clinical trials to confirm their efficacy and safety in human populations. This highlights the need for advanced research, including mechanistic studies and clinical validation, to translate preclinical findings into clinical applications.

**FIGURE 5:**
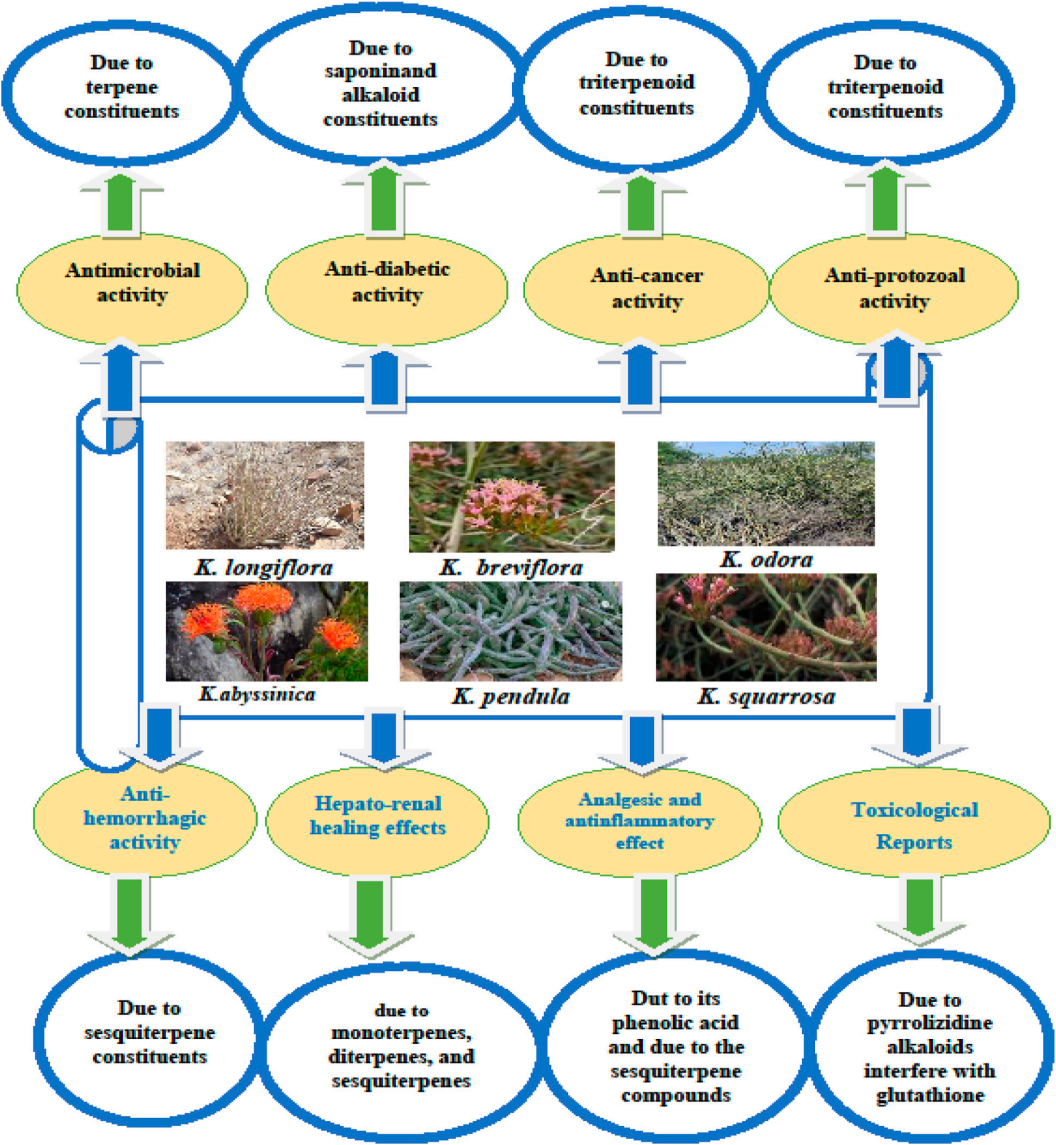
A diagram illustrating key phytochemical metabolites from various Kleinia taxa linked to their pharmacological and toxicological effects, as identified through *in vitro* and *in vivo* screening tests.

This review outlines the primary pharmacological and toxicological activities of various Kleinia taxa, as depicted in Figure 5. Table 6 provides a detailed summary of pharmacological agents, extract types, dosage ranges, controls, and experimental models, offering a systematic perspective on the pharmacological landscape of the *Kleinia* genus and identifying critical areas for further exploration.

**TABLE 6:** Pharmacological activities of extracts and/or fraction of different parts of *Kleinia* taxa.

Activity (s)	Types of extract, fraction, and part of plants	Minimal dose for extract and fraction	Positive Control	Negative control	Types of scientific research	Types of model	Reference
Anti-diabetic	Aqueous extract of stem bark of *K. squarrosa* and *Kleinia longiflora*	50 mg/kg of body weight	Glibenclimide	Saline	*In vivo*	Alloxan- induced diabetic mice	Juma et al. (2015), Abdirahman et al. (2015)
Cytotoxic	Methanolic extract, hexane and chloroform fractions *of K. pendula*	0.07 s μg to 0.19 µg	Doxorubicin	Saline	*In vitro*	Cytotoxic assay	Sewawa (2021)
Analgesic	Chloroform, ethyl acetate, and *n*-hexane fractions o*f K. pendula and K. longiflora*	100 mg/kg	Diclofenac Sodium	Saline	*In vivo*	Eddy hot plate method	Sewawa (2021), Aati et al. (2024b)
Anti-inflammatory	Chloroform and ethyl acetate fractions of *K. pendula, K. grandiflora,* and *K. anteuphorbium*	100 mg/kg	Diclofenac sodium	Saline	*In vivo*	Carrageenan induced paw edema	Sewawa (2021)
Anti-microbial	Essential oil *K. odora*	Not stated	Tetracycline	Saline	*In vitro*	Agar well diffusion	Al-Taweel et al. (2004)

#### 6.1.1 Pharmacological relevance of different *Kleinia* taxa

The genus *kleinia* is of notable pharmacological importance due to the following reasons: Diverse pharmacological and biological activities: *kleinia* species exhibit a wide range of therapeutic properties, including analgesic, anti-inflammatory, anticancer, and antimicrobial effects. These activities address critical medical challenges, such as pain relief, infection control, and chronic disease management. Medicinal value across species: several *kleinia* species, such as *k. Anteuphorbium* and *k. Longiflora*, have demonstrated bioactive properties, highlighting the genus as a valuable source of compounds with potential applications in treating various diseases. Scientific validation: traditional claims about *kleinia*’s medicinal properties have been substantiated through studies using *in vitro* and *in vivo* models, providing essential insights into its biological activities and laying the groundwork for drug discovery. Gaps in mechanistic and clinical validation: despite promising preclinical evidence, detailed studies on the mechanisms of action and clinical trials remain limited. Understanding these mechanisms is crucial for optimizing therapeutic efficacy and minimizing adverse effects. Need for advanced research: addressing existing research gaps, including the lack of clinical evidence, calls for focused pharmacological investigations and translational studies to fully harness *kleinia*’s therapeutic potential. Systematic data representation: visual and tabular representations, such as Figure 5 and Table 6, provide a structured overview of pharmacological agents, experimental models, and dosage data. These summaries help identify research trends and inform future studies.

In conclusion, the *Kleinia* genus holds immense promise for addressing major health challenges. Realizing this potential requires rigorous mechanistic research, clinical validation, and systematic investigations to develop effective and safe therapeutic applications.

#### 6.1.2 Antimicrobial activity

Essential oil of the *K. odora* was subjected to antimicrobial screening using pure strains of *Escherichia coli*, *Staphlococcus aureus*, *and Candida albicans* that were generated from stock cultures in accordance with the general qualitative assay using agar well diffusion assay. Amphotericin is used for fungi and tetracycline for bacteria as positive controls. A twofold serial dilution test was used to find the minimal inhibitory doses for each drug. The oil shows moderate antimicrobial activity against *Echerichia coli* and antifungal activity against *Candida albicans*. This antimicrobial activity of the genus *Kleinia* is due to terpene metabolites (Al-Taweel et al., 2004). Additionally, research verified that *K. anteuphorbium*, formerly known as *Senecio anteuphorbium* Sch. Bip., possesses antimicorbial activity, which is ascribed to its high essential oil concentration (Elhidar et al., 2019).

#### 6.1.3 Anti-diabetic activity

The *in-vivo* antidiabetic effects of aqueous stem bark extracts from *Kleinia squarrosa* and *Kleinia longiflora* were evaluated in both alloxan-induced diabetic mice and control mice. Both oral and intraperitoneal administration of the aqueous extract of *K. squarrosa* and *Kleinia longiflora* showed hypoglycemic activity at four test dose levels (50 mg/kg, 100 mg/kg, 200 mg/kg, and 300 mg/kg of body weight). However, intraperitoneal administration is more effective in lowering blood glucose levels than the oral route. This anti-diabetic activity of the genus *Kleinia* is due to saponin and alkaloid metabolites (Juma et al., 2015; Abdirahman et al., 2015).

#### 6.1.4 Anti-protozoal activity

Petroleum ether and chloroform extracts of *K. odora* and *K. longiflora* show potent activity against *T. brucei* with IC50 values of 0.5 μg/mL. The chloroform extract gives slightly higher selectivity (SI = 63) compared to the petroleum ether extract (SI = 39). The chloroform extract of *K. odora K. abyssinica,* and *K. longiflora* also shows moderate activity against *P. falciparum* schizonts and intracellular amastigotes of *L. infantum* (IC50 of 8 μg/mL). In comparison, the petroleum ether extract showed similar activity against *P. falciparum*, *L. infantum,* and *T. cruzi* (IC50 of 8.6, 6.8, and 5.7 μg/mL), but with lower selectivity. This antiprotozoal activity of the is attributed to triterpenoid metabolites (Al Musayeib et al., 2013).

#### 6.1.5 Anti-hemorrhagic activity

The clotting time assay conducted on petroleum ether extracts from the dry leaves of *K. aizoides* revealed vitamin K activities, which are essential for treating both internal and external hemorrhages. The anti-hemorrhagic effects of *K. aizoides* are found in the fat-soluble and unsaponifiable non-sterol fraction. This activity within the genus *Kleinia* is attributed to its sesquiterpene (like α-caryophyllene) metabolites (Nunez Melendez, 1956).

#### 6.1.6 Cytotoxic activity

The cytotoxic potential of the methanolic extract, as well as the hexane and chloroform fractions of *K. pendula*, was evaluated against breast cancer, liver cancer, and colon cancer cell lines. All tested extracts and fractions of *K. pendula* exhibited significant cytotoxic effects across all three cancer cell lines. The IC50 values of the methanolic extract, hexane and chloroform fractions were comparable to those of the standard chemotherapy drug, doxorubicin. Notably, the hexane fraction showed particularly strong cytotoxic activity, with IC50 values ranging between 0.07 µg and 0.19 µg. This cytotoxic effect of the *K. pendula* is attributed to its triterpenoid and flavonoid metabolites (Sewawa, 2021).

#### 6.1.7 Analgesic, anti-pyretic, anti-inflammatory, and anti-oxidant activities

Phytochemical research shows that essential oils, as secondary metabolites, are produced by plants in response to stress caused by various external factors. These oils are highly valued in the market due to the bioactive properties of their metabolites. Plant-derived phytochemicals, such as terpenes and terpenoids found in essential oils, possess significant pharmacological and biological properties, including antioxidant (55, 67). The chloroform, ethyl acetate, and n-hexane fractions of *K. pendula* and *K. longiflora* were evaluated for their analgesic effects, with dosage levels determined by acute oral toxicity studies. Mice were administered *K. pendula* fractions at doses of 100, 200, and 300 mg/kg body weight, and analgesic activity was measured using Eddy’s hot plate method at 0, 30, 60, and 120 min post-administration. The chloroform, ethyl acetate, and n-hexane fractions of *K. pendula* demonstrated a significant increase in analgesic activity at 30 min, comparable to the effect of the standard drug diclofenac sodium (10 mg/kg). This analgesic effect is attributed to its phenolic acid metabolites (Sewawa, 2021; Aati et al., 2024b). On the other hand, the chloroform and ethyl acetate fractions of *K. pendula, K. grandiflora,* and *K. anteuphorbium* were evaluated for their anti-inflammatory properties in mice treated with standard saline and developed inflammation. Mice administered 100, 200, and 300 mg/kg of these fractions exhibited a significant reduction in inflammation. This anti-inflammatory effect is believed to be due to the sesquiterpene metabolites present in the extracts (Sewawa, 2021). Two-diphenyl-1-picrylhydrazyl (DPPH) free radical scavenging, nitric oxide scavenging, and ferric reducing/antioxidant power (FRAP) assays have revealed that a number of plants that contain essential oils have antioxidant qualities (Karaçelik et al., 2022a; Karaçelik et al., 2022b; Saral et al., 2024).

Studies on *K. anteuphorbium* essential oil have identified potent anti-hyperpyrexia and antioxidant effects, validated through 2, 2-diphenyl-1-picrylhydrazyl (DPPH) free radical scavenging, nitric oxide scavenging, and ferric reducing/antioxidant power (FRAP) assays. It has also proven effective in alleviating pain, inflammation, and fever in various animal models. Its anti-nociceptive properties were assessed via the hot plate, tail-flick, and acetic acid-induced writhing tests. The hot plate test examined the animals’ responses to heat, while the tail-flick test focused on spinal reflexes (Aati et al., 2024b). *K. anteuphorbium* essential oil delayed tail-flick responses, suggesting spinal involvement, and increased reaction times in the hot plate test. It also reduced writhing in rats subjected to acetic acid, indicating both central and peripheral pain-relief mechanisms. Studies showed that monoterpeines with analgesic activity (Guimarães et al., 2013). The analgesic effects are likely linked to active metabolites such as α-pinene, α-terpineol, β-pinene, and myrtenal (Aati et al., 2024b; Park et al., 2010; Chapman et al., 1985). *K. anteuphorbium* essential oil demonstrated anti-fever effects in rats treated with brewer’s yeast, suggesting its efficacy in managing fever through its antioxidant and anti-inflammatory properties (Aati et al., 2024b). Additionally, research has demonstrated that monoterpenes, such α-terpineol, have anti-inflammatory and antioxidant qualities (Sales et al., 2020).

#### 6.1.8 Hepatic and renal protective activities

Treatment with *K. anteuphorbium* essential oil restored liver function markers to normal levels, especially at the higher dose of 10 mg/kg BW, compared to the carbon tetra chloride-treated group. *K. anteuphorbium* essential oil also balanced lipid profile markers during carbon tetra chloride exposure. Additionally, co-administration of *K. anteuphorbium* essential oil with carbon tetra chloride reduced the harmful effects on kidney function markers like urea, uric acid creatinine, and bringing them back to control levels. The higher dose showed a strong protective effect, highlighting *K. anteuphorbium* essential oil’s hepato-protective potential. This effect is likely due to bioactive metabolites such as α-pinene, α-terpinal, α-gurjunene, caryophyllene oxide, silphiperfol-6-ene, myrtenal, (E)-β-caryophyllene and β-pinene, which belong to monoterpenes, diterpenes, and sesquiterpenes. These metabolites have antioxidant and anti-inflammatory properties known to protect the liver and kidneys (Aati et al., 2024b).

### 6.2 Toxicological activities from the genus *Kleinia* taxa

Due to the widespread availability, affordability, and acceptance of medicinal plants, evaluating their safety and toxicity is essential. Concerns over toxicity are a key reason healthcare professionals hesitate to integrate herbal remedies into formal healthcare. The potential long-term toxicity or mutagenicity of many plant families remains underreported, highlighting the need for thorough screening to separate harmful effects from therapeutic benefits (Odeyemi and Bradley, 2018).

The long-term toxic or mutagenic effects of many plant families are still insufficiently studied. This highlights the need for thorough testing to distinguish harmful effects from therapeutic ones. When scientific proof of a plant’s safety is lacking, traditional knowledge of its use becomes essential. Pyrrolizidine alkaloids are toxic metabolites found in plants, often linked to acute and chronic liver damage. These toxins are a leading cause of plant poisoning in humans, livestock, and other animals globally. Over 660 different types of Pyrrolizidine alkaloids have been identified in approximately 6,000 plant species, with around 120 known to be harmful to the liver. Asteraceae are known to produce pyrrolizidine alkaloids, metabolites with recognized hepatotoxic effects. However, the toxicological characteristics of most *Kleinia* taxa remain largely unclear and require more in-depth investigation (Odeyemi and Bradley, 2018; Langel et al., 2011; Stegelmeier, 2011; Lu et al., 2024; Seremet et al., 2018; Şeremet et al., 2016) except *K. neriifolia* which has been confirmed to contain pyrrolizidine alkaloids (Dominguez et al., 2008).

Pyrrolizidine alkaloids exist in two molecular forms: the non-toxic N-oxide and the free base. N-oxides are the dominant form in plants, but in herbivores’ guts, they are easily broken down into the free base, which enters the bloodstream. When pyrrolizidine alkaloids bind to glutathione, the body becomes less able to neutralize reactive oxygen species, leading to increased oxidative stress and membrane damage. Pyrrolizidine alkaloids are also known to produce genotoxicity and hepatotoxicity due to the formation of reactive pyrrolic metabolites that can attach to nucleophilic substances, such as DNA, and create cross-links between DNA and proteins (Belal and El-Gendy, 2014; Yan et al., 2023; He et al., 2021).

In conclusion, the usage of herbal medicines has grown, and Pyrrolizidine alkaloids poisoning is now regarded as a public health concern. Thus, in order to protect the health of humans and animals, it is essential to increase our understanding of the chemistry, pharmacology, and toxicity of pyrrolizidine alkaloids (Moreira et al., 2018).

## 7 Limitation of the study

While numerous studies have investigated the medicinal potential of *Kleinia* species, much of the evidence stems from traditional knowledge and preclinical research. This highlights the necessity for advanced mechanistic studies and translational research to bridge the gap between preclinical findings and clinical applications. There is a scarcity of well-designed clinical trials to validate its therapeutic efficacy in humans. The chemical composition of *Kleinia* varies depending on environmental factors, plant part used, and extraction methods. This inconsistency poses challenges for standardizing extracts and assessing their pharmacological activities. Toxicity studies on *Kleinia* are still in their infancy. Limited data on long-term safety, dosing thresholds, and potential adverse effects make it difficult to fully assess its toxicological risk. Most studies have focused on a narrow range of species or specific geographic regions, leading to a fragmented understanding of the genus as a whole. Another weakness of this study is the unclear relationship between the extract’s components and bioactivity. Broader investigations are needed to cover more species and regions where *Kleinia* is used traditionally.

## 8 Future directions

Future research should prioritize conducting randomized, placebo-controlled clinical trials to determine the safety, efficacy, and appropriate dosing of *Kleinia* species for various therapeutic applications. Developing standardized extraction techniques and identifying bioactive metabolites across different *Kleinia* species are critical for ensuring consistency in therapeutic formulations. More comprehensive studies, including chronic toxicity, genotoxicity, and long-term safety evaluations, are necessary to better understand the toxicological profile of *Kleinia* and establish safe usage guidelines. Expanding ethnobotanical surveys and phytochemical studies across a broader range of *Kleinia* species and geographic regions could uncover additional medicinal applications and unique metabolites. Investigating the molecular mechanisms behind the pharmacological activities of *Kleinia* species will provide insight into their potential therapeutic benefits and guide future drug development. Research into sustainable harvesting practices is essential to preserve *Kleinia* species, especially in regions where it is over-exploited, while promoting conservation efforts and responsible use of its medicinal resources. By addressing these limitations and focusing on these research priorities, the therapeutic potential of *Kleinia* can be fully explored and safely integrated into modern medicine.

## 9 Conclusion

In conclusion, *Kleinia* (Asteraceae) holds immense potential in the field of medicinal research due to its diverse phytochemical metabolites and pharmacological properties. This review highlights the genus’ rich array of secondary metabolites that exhibit a wide range of biological activities, such as anti-microbial, anti-protozoal, anti-hemooragic, analgesic, anti-pyretic, anti-inflammatory, anti-diabetic, cytotoxic, and antioxidant effects. However, despite its promising therapeutic potential, much of *Kleinia’s* medicinal value remains underexplored, with only a limited number of species investigated to date. The presence of potentially toxic metabolites, notably pyrrolizidine alkaloids, underscores the importance of cautious application in traditional medicine and the need for comprehensive toxicological evaluations. Expanded phytochemical research is vital to uncover additional bioactive metabolites, and further preclinical and clinical trials are needed to establish the efficacy and safety of *Kleinia* extracts. To maximize the therapeutic potential of this genus, interdisciplinary collaboration among researchers, pharmaceutical developers, and traditional medicine practitioners is essential. By addressing these research gaps and fostering partnerships, *Kleinia* can be developed into safe, effective, and sustainable medicinal products that contribute to modern healthcare.
